# Public Awareness and Barriers to Seeking Medical Advice for Colorectal Cancer in the Gaza Strip: A Cross-Sectional Study

**DOI:** 10.1200/JGO.18.00252

**Published:** 2019-05-03

**Authors:** Mohamedraed Elshami, Maha Alfaqawi, Tamer Abdalghafoor, Ayoob A. Nemer, Mohammed Ghuneim, Hussien Lubbad, Batool Almahallawi, Mosab Samaan, Abdallah Alwali, Ahmad Alborno, Deyaa Al-kafarna, Aseel Salah, Karam Shihada, Mohammed Abo Amona, Amira Al-Najjar, Rana Abu Subha, Basma Alhelu, Israa Abujayyab, Loai Albarqouni, Bettina Bottcher

**Affiliations:** ^1^ Ministry of Health, Gaza, Palestine; ^2^ Islamic University of Gaza, Gaza, Palestine; ^3^ Bond University, Queensland, Australia

## Abstract

**PURPOSE:**

Raising awareness of colorectal cancer (CRC) symptoms for early recognition, reduction of modifiable risk factors, and removing barriers to seeking medical help could lower its mortality. This study aimed to assess the level of public awareness of CRC in the Gaza Strip.

**MATERIALS AND METHODS:**

This was a cross-sectional study conducted at three hospitals and 10 high schools between September and October 2017. The Arabic version of the validated Bowel Cancer Awareness Measure (BoCAM) questionnaire was used to evaluate awareness of CRC symptoms and risk factors, and barriers to seeking medical help. Adults (age ≥ 18 years) in three major hospitals and adolescents (ages 15 to 17 years) in 10 schools were recruited for face-to-face interviews to complete the BoCAM.

**RESULTS:**

Of 3,172 potential participants, 3,080 completed the BoCAM (response rate, 97.1%). Among these, 1,578 (51.2%) were adults and 1,614 (52.4%) were females. Persistent abdominal pain was the most commonly recognized CRC symptom (n = 1,899; 61.7%), whereas anorectal pain was the least common (n = 1,056; 34.3%). In total, 2,177 (70.7%) were not confident in recognizing CRC symptoms or signs. Having a bowel disease was the most frequently recognized CRC risk factor (n = 1,456; 47.3%) and diabetes the least recognized (n = 591; 19.2%). The overall mean scores ± standard deviations for recalling and recognizing CRC symptoms were 1.2 ± 1.3 and 4.3 ± 2.3, respectively (out of 9 points). The overall mean scores ± standard deviations for recalling and recognizing CRC risk factors were 0.7 ± 0.8 and 8.0 ± 3.1, respectively (out of 16 points). Emotional barriers were the most commonly reported barriers to seeking medical help, with feeling worried about what a doctor might find as the most common barrier (n = 1,522; 49.4%).

**CONCLUSION:**

Public awareness of CRC is suboptimal in Gaza. Improving CRC awareness with educational interventions is needed, including in local schools.

## INTRODUCTION

Globally, colorectal cancer (CRC) is the third most common malignancy and the fourth most frequent cause of cancer-related deaths.^1^ In Gaza, CRC is the most common cancer among males, accounting for 15.5% of their cancers, and second to breast cancer in women, accounting for 11.2% of their cancers.^2^ This is higher than a worldwide estimate of 10.6% of CRC among all patients with cancer in 2018.^3^ It also has incidence rates of 11.5 and 10.3 per 100,000 of male and female populations, respectively, in Gaza and is the second most frequent cause of cancer-related deaths, responsible for 11.0% of total cancer-related deaths.^4^ Such high mortality rates could be a result of diagnosis at advanced stages due to low awareness levels of CRC symptoms and risk factors, and difficult access to health care facilities.

Greater public awareness of CRC symptoms may lead to less delay before seeking medical advice that, in turn, will facilitate early detection of CRC, increase survival rates, and improve outcomes.^5-7^ Furthermore, the lack of a CRC screening program in Gaza necessitates raising CRC awareness among the general population.^2^

**CONTEXT**

**Key Objective**
The increasing incidence and high mortality rates of colorectal cancer (CRC) in the Gaza Strip make it an important public health concern. Therefore, this study examined public awareness of symptoms and risk factors, as well as reported barriers to seeking medical help and compared these between men and women, as well as adults and adolescents.
**Knowledge Generated**
Poor public knowledge of CRC symptoms and risk factors, as well as the other reported barriers found in this study, may play a significant role in the diagnosis of CRC at advanced stages because of delays before patients see the doctor, ultimately leading to a lower survival rate.
**Relevance**
A systematic national education program to promote the public awareness of CRC tailored to suit all age groups is needed. In addition, an urgent need to establish a CRC screening program to facilitate its early detection exists.

Generally, women are believed to display more health-related behaviors than men in Palestine. However, recent studies have shown increasing smoking rates among female university students and higher obesity rates among women.^8,9^ Moreover, a previous study on breast cancer awareness in Gaza showed significantly higher awareness among adult women, compared with adolescent females,^10^ despite health education being part of the school curriculum. Exploring the health awareness of adolescents on a variety of issues is important because this might shape their health-related behavior in the future. In view of the high proportion of young people in the Palestinian population, with 39% younger than 15 years of age and 30% 15 to 29 years of age, it is an important long-term investment.^11,12^ Younger age groups (15 to 24 years and 25 to 34 years) represent 2.5% and 5.2%, respectively, of the total reported patients with CRC from 2009 to 2014 in Gaza.^13^

This study aimed to explore (1) public awareness of CRC symptoms and risk factors in Gaza, (2) public awareness of CRC age-related risk, (3) the potential barriers to seeking medical help, and (4) differences between population groups, such as men and women, as well as adults and adolescents.

## MATERIALS AND METHODS

### Study Design and Population

This was a cross-sectional study conducted from September 1 to October 31, 2017, using the Bowel Cancer Awareness Measure (BoCAM) questionnaire, which is a validated measurement for public awareness of CRC.^14^ Awareness levels were compared among different population groups, such as between men and women and between adolescents and adults. The questionnaire consists of five sections: (1) demographic data; (2) evaluation of knowledge of age-related CRC risk and confidence to detect its symptoms; (3) open-ended (recall) questions and (4) closed (recognition) questions with a comparison between the outcomes using both recall versus recognition; and (5) barriers to seeking medical advice. A 3-point scale, with answers yes, no, and I do not know, was used to evaluate the recognition of signs and symptoms of CRC, as well as to explore barriers to seeking medical help, that were further categorized into emotional, practical, and service barriers. A 5-point Likert scale was used to assess the recognition of CRC risk factors.

The BoCAM was translated from English to Arabic and then back-translated into English by several people proficient in both languages. Before starting data collection, a pilot study was conducted with 92 respondents to test the clarity of the questions of the Arabic version of BoCAM. A reliability analysis was carried out on the perceived task values scale comprising 29 items. Cronbach’s alpha (0.72) showed that the questionnaire reached acceptable reliability. Although it has not been validated, a similar questionnaire was used in some previous studies conducted in Arabic-speaking countries.^11,15,16^

### Sampling Methods

Health care services in the Gaza Strip are provided by the government, nongovernmental organizations, or private providers. Governmental hospitals are the main entry point for health care services in Gaza because they provide most basic health care at no or little cost to the insured population.^10^ Health care insurance is obtainable at low cost. Nongovernmental organization facilities often provide specialized health care in certain areas, such as burn care or limb reconstruction. The fees of private hospitals prohibit most people from accessing these services. Therefore, men and women 18 years of age or older admitted to or visiting governmental hospitals were the target population to get a broad representation of the general population. Patients or visitors to oncology departments were excluded from the study.

There are 13 governmental hospitals in Gaza.^2^ From these, the largest three, located in separate geographic locations, were chosen for recruitment of participants by stratified sampling. This sampling area covered most of Gaza’s population, producing a representative sample. Parallel to this, adolescents from 10 high schools (out of 147^17^), located in the same areas as the study hospitals, were recruited. High school students study health-related topics in their curriculum, which presented the opportunity to explore their awareness of CRC. Participants were invited for face-to-face interviews to complete the BoCAM.

Data collectors were trained to recruit participants, distribute the questionnaires, and facilitate completion. Before completing the questionnaire, a detailed explanation of the study, including its purpose, was given to the participants. Informed consent was obtained from the participants, and ethical approval was obtained from both the Palestinian Ministry of Health and the Ministry of Higher Education.

### Statistical Analysis

Descriptive statistics were used to report the knowledge of age-related CRC risk. One unprompted open question and nine prompted closed questions assessed the knowledge of CRC signs and symptoms. The unprompted question asked participants to write down the CRC signs and symptoms they could remember, whereas the closed questions assessed knowledge on specific signs and symptoms. Every correctly recalled sign/symptom or correct answer in the closed questions (yes) was given 1 point, whereas incorrect answers (no and I do not know) received no points.

Another open question requested recall of CRC risk factors, and eight closed questions assessed recognition of CRC risk factors. Every correctly recalled risk factor was given 2 points. Answers to the eight closed questions were scored on a 5-point Likert scale. This was converted to a 3-point scale, because it was difficult for participants to distinguish between agree versus strongly agree and disagree versus strongly disagree; therefore, the response strongly agree was recoded to agree, and strongly disagree was recoded as disagree.^10^ Disagree was given no points, not sure was given 1 point, and agree was given 2 points. Cumulative scores were calculated for recognizing CRC signs and symptoms as well as risk factors and reported as mean ± standard deviation out of the total score of 9 for signs and symptoms and 16 for risk factors. Furthermore, 10 questions were asked about barriers to seeking medical advice that were scored yes, no, and I do not know, and are reported as total numbers and percentages for each point.

The variable of interest was the overall awareness mean score for each section (signs/symptoms and risk factors), for which the one-sample *t* test was used. The two-sample *t* test was used to compare the total mean scores of recall and recognition and their percentages between male and female as well as adult and adolescent participants, which were normally distributed. The χ^2^ test was used to compare the awareness of each CRC symptom and risk factor between these two subpopulations. Multiple logistic regression was used to test the association between sex and age group with recalling CRC symptoms and risk factors. It was also used to test their association with recognizing the symptoms and to test the relationship between this recognition and having barriers to seeking medical advice. Ordinal regression was used to test the association of age group and sex with recognizing risk factors. Data were analyzed using Stata software version 15.0 (StataCorp, College Station, TX).

## RESULTS

### Characteristics of Participants

Of 3,172 invited participants, 3,080 completed the BoCAM questionnaire (response rate, 97.1%). Among these, 1,578 (51.2%) were adults, 1,502 (48.9%) were adolescents, and 1,614 (52.4%) were females. The mean age of all participants was 25.4 ± 12.1 years.

### Knowledge and Confidence to Detect CRC Symptoms

A total of 442 participants (14.4%) were not confident at all about their ability to detect a symptom of CRC, whereas 1,735 (56.3%) were not confident. Generally, awareness of CRC signs and symptoms, as well as risk factors, was low when recall questions were used and higher with recognition questions. Abdominal pain was the most commonly recognized CRC symptom (n = 1,899; 61.7%), whereas anorectal pain was the least common (n = 1,056; 34.3%; Table 1). The overall mean scores for recalling and recognizing CRC symptoms were 1.2 ± 1.3 and 4.3 ± 2.3, respectively, out of 9 possible points. Adults demonstrated higher awareness than adolescents (4.9 ± 2.3 *v* 3.8 ± 2.0 out of 9; *P* < .001). This was also true after adjusting for sex, where adults generally showed a significantly higher likelihood of recalling and recognizing CRC signs and symptoms, although they were less likely to recall abdominal pain (odds ratio [OR], 0.79; 95% CI, 0.69 to 0.92; *P* = .002), and there were no significant associations with recall of anorectal pain and abdominal mass (Table 2). Females had a significantly higher mean score than males (4.5 ± 2.3 *v* 4.2 ± 2.3 of 9; *P* < .001). However, after adjustment for age group, there was no independent association of sex with the recalled CRC signs and symptoms except anorectal pain, where females had a 57% decrease in the odds (OR, 0.43; 95% CI, 0.29 to 0.64; *P* < .001).

**TABLE 1:**
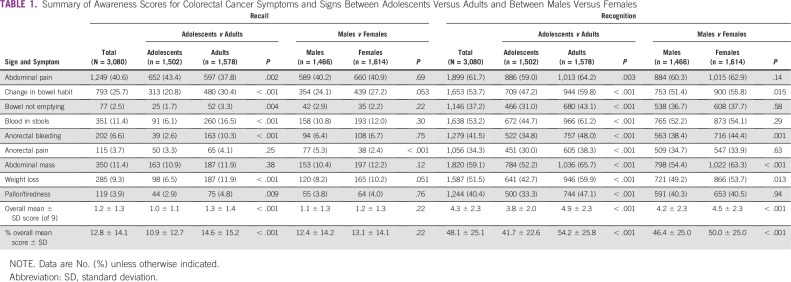
Summary of Awareness Scores for Colorectal Cancer Symptoms and Signs Between Adolescents Versus Adults and Between Males Versus Females

**TABLE 2:**
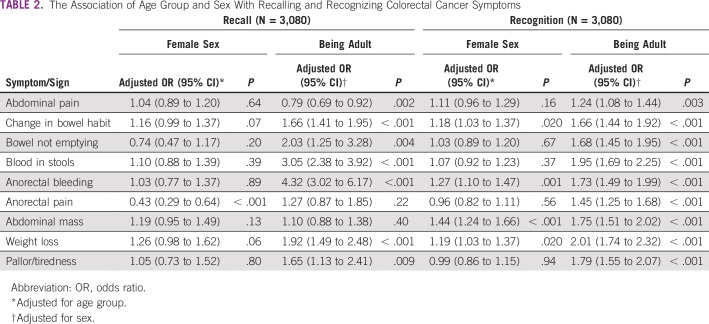
The Association of Age Group and Sex With Recalling and Recognizing Colorectal Cancer Symptoms

### Awareness of CRC Risk Factors

Having bowel disease was the most frequently recognized CRC risk factor (n = 1,456; 47.3%), and diabetes was the least recognized (n = 591; 19.2%; Table 3). Only 918 participants (29.8%) gave a correct answer for CRC age-related risk, whereas 1,391 (45.2%) believed that it was unrelated to age. Out of 16 points, the overall mean scores for recalling and recognizing CRC risk factors were 0.7 ± 0.8 and 8.0 ± 3.1, respectively. Adults demonstrated better recognition of every risk factor and a higher overall score compared with adolescents (8.7 ± 3.2 *v* 7.3 ± 2.8 of 16; *P* < .001). This was also evident after adjusting for sex, except for doing less physical activity, which did not have an association with age group. Females also had significantly higher awareness than males (8.3 ± 3.0 *v* 7.8 ± 3.2 of 16; *P* < .001). However, after adjustment for age group, females had significantly lower odds of recalling eating red or processed meat once a day or more (OR, 0.62; 95% CI, 0.48 to 0.79; *P* < .001) and having bowel disease (OR, 0.69; 95% CI, 0.52 to 0.92; *P* = .010; Table 4). In contrast, females had significantly higher odds of recognizing fiber-free diet (OR, 1.55; 95% CI, 1.36 to 1.76; *P* < .001), having a relative with CRC (OR, 1.42; 95% CI, 1.25 to 1.62; *P* < .001), and having bowel disease (OR, 1.25; 95% CI, 1.09 to 1.43; *P* = .001).

**TABLE 3:**
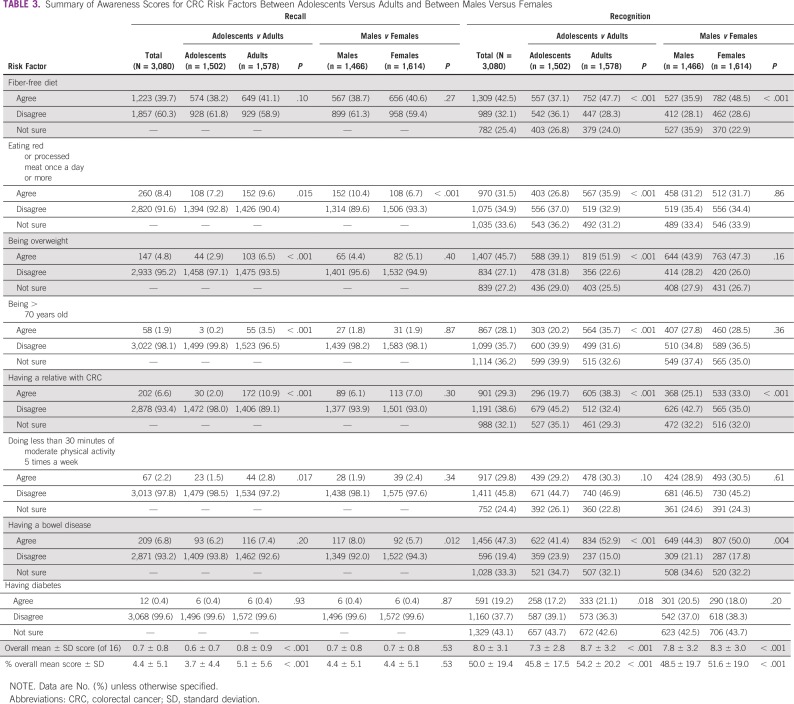
Summary of Awareness Scores for CRC Risk Factors Between Adolescents Versus Adults and Between Males Versus Females

**TABLE 4:**
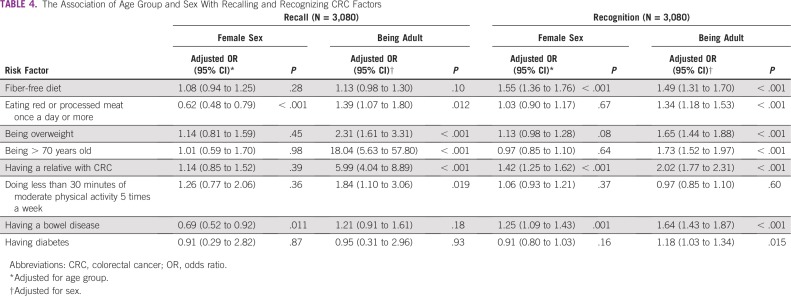
The Association of Age Group and Sex With Recalling and Recognizing CRC Factors

### Barriers to Seeking Medical Advice

Overall, emotional barriers were the most commonly reported barriers to seeking medical help, with feeling worried about what a doctor might find as the most common barrier (n = 1,522; 49.4%; Table 5). This was also found among adults (n = 773; 49.0%) and females (n = 859; 53.2%).

**TABLE 5:**
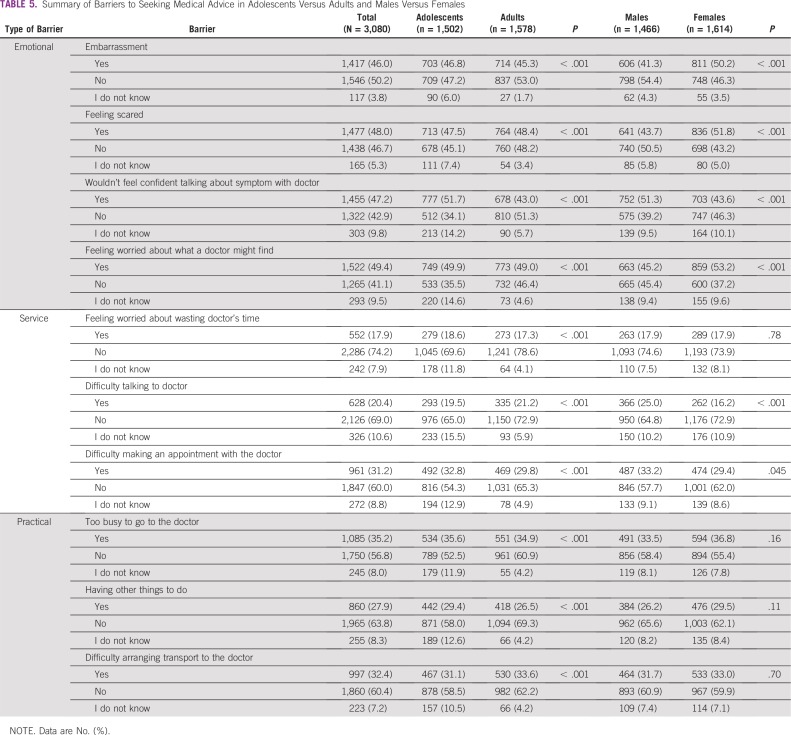
Summary of Barriers to Seeking Medical Advice in Adolescents Versus Adults and Males Versus Females

However, insecurity in talking about CRC symptoms with a doctor was the most frequent barrier among adolescents (n = 777; 51.7%) and males (n = 752; 51.3%). Tables 6 and 7 list the relationships between recognizing CRC symptoms and risk factors and reporting a barrier to seeking medical advice.

**TABLE 6:**
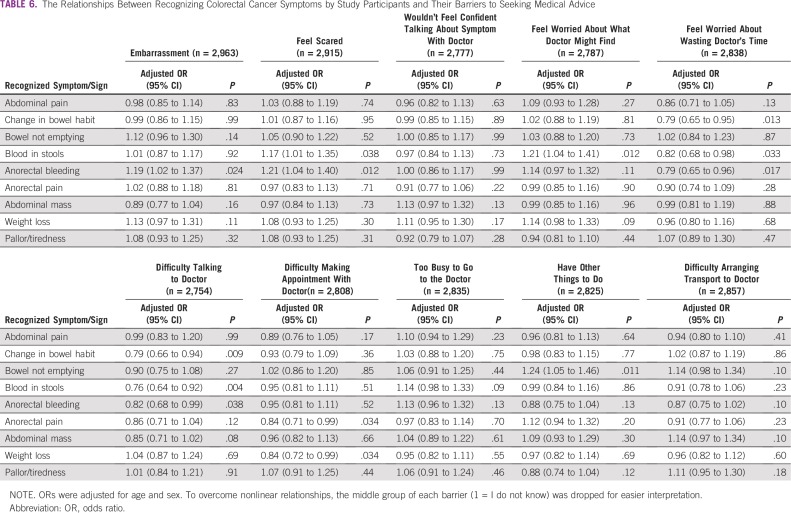
The Relationships Between Recognizing Colorectal Cancer Symptoms by Study Participants and Their Barriers to Seeking Medical Advice

**TABLE 7:**
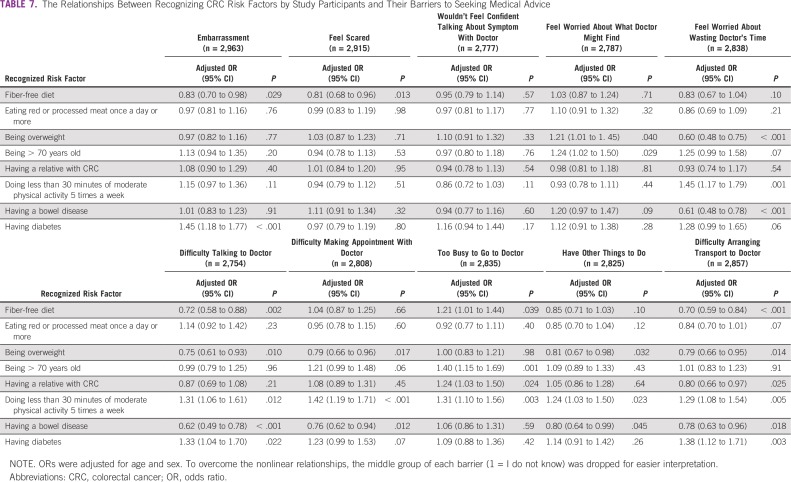
The Relationships Between Recognizing CRC Risk Factors by Study Participants and Their Barriers to Seeking Medical Advice

## DISCUSSION

CRC awareness in Gaza was found to be low. Adults displayed higher awareness than adolescents, and females demonstrated better knowledge than males. Emotional barriers were most commonly reported among the different groups. Insecurity in talking about CRC symptoms with a doctor was the most frequent barrier among adolescents and males, and concern about what a doctor might find was the most frequent barrier among adults and females.

The higher level of CRC awareness among women in this study is consistent with findings from previous studies.^18-20^ Women are in contact with health care services more often than men as a result of pregnancy, family planning, and childcare, and this might promote their health-related knowledge and encourage them to have more protective behaviors than men.^21^

Similar to other studies,^18,19^ adults in this study displayed a better awareness than adolescents. A reason for this may be higher education levels achieved by adults and experiences enabling them to recognize CRC signs and symptoms. Another factor could be that adults were recruited from hospitals and displayed a degree of health-seeking behavior, which might contribute to their greater knowledge.^11,22^ Therefore, targeting young people with educational interventions on modifiable risk factors and alarming symptoms could be especially beneficial. Previous studies conducted in Britain and Jordan found similar low cancer awareness among university students and adolescents.^11,23^ Kyle et al^24^ reported that a school-based educational intervention program was effective in sustainably raising cancer awareness among adolescents. Therefore, cancer awareness—especially of common cancers like CRC—should receive more attention in the school curriculum, because it could have a potential lifelong effect on encouraging early diagnosis.^11,23^ In addition, Power and Wardle^25^ showed that awareness campaigns targeting adults could increase their awareness of CRC symptoms, thus reducing their time to seek medical advice. The lack of recognizing CRC symptoms in 51.9% of participants in this study is comparable to findings from other Arab countries, with 59.0% of Lebanese participants displaying poor knowledge,^26^ 41.0% of participants displaying poor knowledge about CRC symptoms in Bahrain,^16^ 2.8% of participants in Saudi Arabia correctly recognizing CRC symptoms,^27^ and 14.3% of Jordanian university students displaying poor knowledge.^11^ This demonstrates poor knowledge of CRC symptoms in the region, which might be further compounded by a culture of not talking about symptoms that might be perceived as embarrassing, and this assumption is supported by the large proportion of participants reporting embarrassment as a main barrier in this study.

Abdominal pain was the most commonly recognized symptom, as in other studies, which could be attributed to its interference with daily activities.^11,23,28^ However, pallor/fatigability was the most recognized symptom in an Omani study,^15^ with 55.1% recognizing the symptom compared with 40.4% in Gaza. This difference could be caused by the comparably high prevalence of anemia in Gaza, with rates of 60.3% in patients with heart disease,^29^ 35.8% among female adolescents,^30^ and 33.1% among pregnant women,^31^ indicating that anemia is not normally recognized as a CRC sign.^32^

Recognition of blood in stools as a CRC symptom by 53.2% in this study was comparable to 53.0% in the Omani study,^15^ 50.1% of the Jordanian undergraduate students,^11^ and more than the 22.5% reported in a Spanish study.^33^ This underlines the finding that people in Gaza are more alarmed by the obvious symptoms and signs of CRC, whereas common symptoms, such as pallor, and common deficiencies, such as anemia, are not always regarded as abnormal or unusual.

Al-Azri et al^15^ reported a higher recognition among Omani participants than among those from Gaza for CRC risk factors, such as doing less physical exercise (37.3% *v* 29.8%), having a relative with CRC (32.7% *v* 29.3%), and diabetes (24.9% *v* 19.2%). However, Gazans identified the low-fiber diet more frequently (42.5%) than people in Oman (38.7%) and Spain (29.5%).^15,33^

The Omani participants reported a mixture of practical and emotional barriers as the most common barriers to seeking medical advice for CRC.^15^ However, despite the poor economic circumstances in Gaza, emotional barriers were most commonly reported, not service or practical barriers, as would be expected, and higher percentages were obtained especially among females. A possible explanation for this could be that women tend to display a fear of cancer, denial, and reliance on alternative therapies.^23,34,35^ The lack of female oncologists and surgical specialists in Gaza could be another reason, especially in the younger age groups, as found by Elshami et al,^10^ where feeling embarrassed was the most common barrier to seeing a doctor by female adolescents. This was also observed among American women who reported delays in seeking care due to a perceived lack of female clinicians.^36^ However, a study on CRC screening in the West Bank, Palestine, showed similar rates of embarrassment among men and women,^22^ which were also significantly lower, at 11.0% and 11.4%, than those in this study, at 41.3% and 50.2%, respectively. Higher numbers of female doctors and cultural differences might be the reason. This demonstrates the urgent need for more female surgeons and oncologists in Gaza. In addition, men and adolescents in Gaza did not feel confident talking about their symptoms to the doctor, reflecting poor doctor-patient relationships and leading to additional delays in presentation. Poor communication skills by health care professionals have also been shown to affect health care services in other studies from Gaza.^10,37^ Therefore, it is essential to systematically include communication skills and professionalism in undergraduate and postgraduate training in Gaza to make services more accessible, especially to younger people.

The strengths of this study are the large sample size, the high response rate, and the use of a validated instrument, the BoCAM. In addition, the inclusion of both adults and adolescents provides the opportunity for additional recommendations on prevention interventions.

Limitations of this study include the lack of sociodemographic data, such as level of education, that can influence the awareness of CRC. In addition, no additional exploration was performed on how much impact factors such as family history of CRC and familiarity with the disease through friends and neighbors had on participants' knowledge of the disease. Moreover, recruitment of adult participants from hospitals might have caused a degree of selection bias because they displayed health-seeking behavior, which adolescents, recruited from schools, did not.

In conclusion, poor public awareness of CRC symptoms was demonstrated, especially if symptoms were not affecting daily activities. In addition, the potential impact of some modifiable risk factors (such as obesity, lack of physical exercise, and Western diet) on increasing the risk of CRC was poorly understood. Interventions to improve public awareness of CRC, such as educational interventions in schools and the public domain, are warranted and should be tailored to each age group. Emotional barriers, especially among women, should be addressed by training more female clinicians and improving communication skills of existing physicians. Finally, a strategy to establish a CRC screening program in Gaza should be developed to facilitate early detection of CRC in the face of its increasing incidence.
